# Increasing Face Mask Wearing in Autistic Individuals Using Behavior Analytic Interventions: A Systematic Review and Meta-analysis

**DOI:** 10.1007/s10803-023-06128-x

**Published:** 2023-09-26

**Authors:** Rebekah Cowell, Athanasios Vostanis, Peter E. Langdon

**Affiliations:** 1 Tizard Centre, University of Kent, Cornwallis North East, Canterbury, Kent, CT2 7NF UK; 2 Centre for Research in Intellectual and Developmental Disabilities (CIDD), University of Warwick, Coventry, CV4 8UW UK; 3 Coventry and Warwickshire Partnership NHS Trust, Coventry, CV6 6NY UK

**Keywords:** Mask-Wearing, Autism, Meta-Analysis, Hygiene, Tolerance Training

## Abstract

The current review aimed to evaluate the effectiveness of behavior-analytic procedures in increasing face mask-wearing in autistic individuals. This comes following recommended guidance during the COVID-19 pandemic. A systematic review and meta-analysis were completed of peer-reviewed and grey literature. Six databases were searched and seven studies using single-case experimental designs met the eligibility criteria which were then quality appraised. Data were extracted on participant characteristics, study design, independent and dependent variables, fidelity, generalization, maintenance, and social validity outcomes. Both the non-overlap of all pairs and Baseline Corrected TAU were used to estimate effect size. Two studies were rated strong and borderline strong quality and five were rated as adequate or below. All studies showed positive outcomes for mask-wearing, with an average of 0.92 for non-overlap of all pairs and 0.47 for Baseline Corrected Tau effect sizes. The most common and effective procedures for increasing mask-wearing were graded exposure and differential and positive reinforcement. Factors such as mode of delivery, implementer, and setting did not appear to influence study outcomes. Procedures were found to be rated as acceptable by parents and professionals in five of the studies. The existing literature on increasing face mask-wearing in autistic individuals provides promising findings to add to existing literature around increasing tolerance to medical equipment and hygiene practices in autistic populations. However, these findings are based on a small sample size, with six of the studies taking place in the United States with varying study quality.

## Introduction

The novel coronavirus (COVID-19) outbreak was declared a global pandemic by the World Health Organization in March 2020 (World Health Organization [WHO], 2020a). SARS-CoV-2 is a virus that affects multiple organ systems, including the lungs, heart, kidneys, and brain. The respiratory symptoms produced can be life-threatening, particularly in vulnerable populations or those with pre-existing medical conditions (Lake, 2020; Wang et al., 2020; Zhou et al., 2020). SARS-CoV-2 primarily transmits via air droplets, and individuals can spread the virus even if they are asymptomatic or have mild symptoms (Howard et al., 2021). Several measures were implemented worldwide to reduce the spread, such as lockdowns, face masks, social distancing, travel restrictions, and contact tracing (Esposito & Principi, 2020; Güner et al., 2020; WHO, 2022b). These measures applied to children, adolescents, and adults. Although children and adolescents tend to have more mild or asymptomatic presentations of COVID-19, evidence has shown that the viral load carried by them is the same of an adult, supporting the notion that all should wear face masks (Esposito & Principi, 2020; Howard et al., 2021) Existing evidence suggests in both laboratory and clinical settings that face mask-wearing reduces transmission of infected respiratory droplets and spray (Howard et al., 2021; Lio et al., 2021). It has also been found that masks effectively minimize viral spread in health and public settings (Chu et al., 2020).

Several risk factors for COVID-19 have been identified including age, sex, and underlying medical needs such as diabetes and cardiovascular disease (Rashedi et al., 2020). Autistic individuals have been found to be more vulnerable to COVID-19 due to associated modifications within their immune systems (Lima et al., 2020). Autistic individuals are more vulnerable to illness as they are less likely to engage in good hygiene practices such as hand washing, refraining from face touching, and wearing face masks (Halbur et al., 2021, 2022; Sivaraman et al., 2021). They are also more likely to display behaviors described as challenging, maintained by escape and avoidance around wearing medical protective equipment such as face masks (Sivaraman et al., 2021). Wearing a mask may be an unfamiliar or uncomfortable experience, particularly for autistic individuals who experience a delay in adaption to novel sensations or have heightened sensitivity to touch. As a result, mask-wearing might potentially be an aversive experience (Allen & Kupzyk, 2016; Puts et al., 2014; Slifer et al., 2008). Furthermore, autistic individuals often have social and communication difficulties, which may impact their ability to recognise and communicate signs of illness (e.g., pain, breathing issues) to gain the appropriate medical treatment (Lillie et al., 2021). Another key risk factor for autistic individuals is that there may be difficulty in understanding what COVID-19 is and its impact due to this being an abstract concept, making it more difficult for some of them to follow and benefit from COVID-19 preventative measures (Mutluer et al., 2020). This fact is particularly important as recent studies have shown that individuals with intellectual and developmental disabilities (IDD) have a higher risk of death from COVID-19 and are 2.5 to 4 times more likely to contract SARS-Cov-2 than their typically developing peers (Shapiro, 2020).

A number of studies over the years have successfully applied behavioral interventions to increase autistic people’s, and primarily children’s, tolerance of medical equipment and procedures such as prescription glasses (DeLeon et al., 2008), routine physical medication examinations (Gillis et al., 2009), magnetic resonance imaging (MRI) (Cox et al., 2017), medical routines (Allen & Kupzyk, 2016), medical bracelets (Cook et al., 2015), foot orthopedics, and hearing aids (Richling et al., 2011). A literature review by Allen and Kupzyk (2016) found that contingent reinforcement and graded exposure are the most common behavioral interventions used to help individuals overcome fear or avoidance of medical procedures. Other commonly used intervention components were escape extinction, modeling, prompting, and behavioral momentum.

DeLeon et al. (2008), successfully increased prescription glasses wearing for four individuals with intellectual disabilities through noncontingent reinforcement (NCR), response cost, and brief response blocking. Building on these findings, Richling et al. (2011) used NCR to increase compliance with foot orthopedics and hearing aids without response blocking with two participants. They found that NCR successfully increased tolerance for wearing the equipment from zero minutes in baseline to wearing these three hours post-intervention for both participants, and generalization was achieved across different settings. Although many studies include the use of response blocking, it is not always ethical or practical and may evoke challenging behavior. Therefore, many studies have incorporated differential reinforcement of other behavior (DRO) as an effective alternative to increase cooperation without blocking (Dufour & Lanovaz, 2019).

Cook et al. (2015), implemented differential negative reinforcement of other behavior (DNRO) using a changing criterion design to increase an autistic boy’s tolerance of wearing a medical bracelet. They found that the duration of wearing the bracelet could be extended from five seconds to seven hours over several weeks, and the participant continued to wear this over the next two years following the study. Furthermore, Dufour and Lanovaz (2019) replicated prior research by evaluating the use of DRO without extinction to increase tolerance of medical devices, specifically a heart rate monitor. They found that tolerance to the device increased to 100% when receiving a reinforcer every 90 s for both participants. However, a limitation of this was the terminal criterion of 90 s not being sustainable for devices to be worn over a prolonged period. In another recent paper, Cox et al. (2017) increased tolerance to MRI scans using a mock MRI machine across two studies. The first study used a combined intervention of stimulus fading, prompting and contingent reinforcement while the second study used prompting and DRO without extinction. Results successfully generalized for three of the seven participants who tolerated a real MRI scan.

Given the evidence surrounding the COVID-19 pandemic and recommendations to reduce the spread of the virus, a review of the available evidence is important to evaluate the methods used to increase face mask-wearing in this population, which has not been completed to date. Whilst COVID-19 regulations have now been widely discontinued, mask wearing continues to be a key measure alongside other hygiene practices to prevent the spread of COVID-19 and other illnesses in circumstances such as medical and community settings when actively unwell and to protect medically vulnerable populations (Wang et al., 2020). The current paper aims to complete a systematic review and meta-analysis of the literature looking to increase face mask-wearing in autistic individuals using behavioral interventions. The research questions to be explored in this review were: (a) What are the main intervention components used to increase face mask-wearing in autistic individuals? (b) How socially valid are the procedures used to increase face mask-wearing in autistic individuals? (c) What are the most effective procedures to increase face mask-wearing in autistic individuals? (d) What factors influence the effectiveness of these procedures?

## Method

### Eligibility Criteria

Empirical studies available in English were included, without any restrictions placed on the publication year, if they met the following criteria:

1. Participants were autistic.
2. The intervention was behavior-analytic. Each study was evaluated against all seven dimensions of applied behavior analysis (ABA) as proposed by (Baer et al., 1968; see Table 1).
3. Outcomes included a measure of behavior related to face mask-wearing.
4. Studies included a baseline to intervention comparison for single-case experimental designs or pre- to post-intervention comparison for group studies.

**Table 1:** Final Screening Against the Seven Dimensions of Applied Behavior Analysis

	Generality	Effective	Technological	Analytic	Conceptually Systematic	Applied	Behavioral
Aaronson et al. (2021)	**No.** No generalization or maintenance probes or procedures described.	**Unclear.** No prior data on mask wearing behaviors to compare to, to assess effectiveness.	**No.** Limited detail provided on procedures.	**No.** No functional relation demonstrated as no pre-measure/baseline/comparison group. Correlational study design.	**No.** Procedures and results not explained using behavior analytic principles.	**Yes.** Looking at feasibility of mask wearing in school aged children during COVID-19 pandemic.	**No.** Data collection procedures described in little detail, no clear behavioral definitions or detailed recording methods provided.
Ertel (2020)	**Yes.** Post-intervention probes were conducted in the waiting room and physician office settings	**Yes.** All participants were able to tolerate the mask for at least one hour after treatment.	**Yes.** Procedures are described in enough detail for replication.	**Yes.** Concurrent MBL across participants with 3 tiers to allow for functional relations to be identified.	**Yes.** Procedures and results are described using behavioral principles; positive reinforcement, reinforcement schedules, exposure hierarchy etc.	**Yes.** Aim to increase face mask wearing during COVID-19 pandemic.	**Yes.** Data collection procedures described in such as a breakdown of hierarchy steps and compliance defined.
Ertel et al. (2022)	**Yes.** Probes in multiple settings and maintenance probes completed 1-month post-treatment.	**Yes.** Face mask wearing increased across all participants.	**Yes.** Procedures are described in enough detail for replication.	**Yes.** Concurrent MBL across participants with 3 tiers to allow for functional relations to be identified.	**Yes.** Procedures and results are described using behavioral principles; positive reinforcement, reinforcement schedules, exposure hierarchy etc.	**Yes.** Aim to increase face mask wearing during COVID-19 pandemic.	**Yes.** Clearly defined behavior data recording procedures (no behavioral definitions included in the paper – prior functional analysis completed).
Frank-Crawford et al. (2021)	**Unclear.** Component analysis completed with 3 participants post-treatment phase taking additional data points to assess maintenance of mask wearing when specific intervention components are removed.	**Yes.** Face mask wearing increased for all participants compared to baseline, only one did not meet the terminal duration.	**Yes.** Procedures are described in enough detail for replication.	**Yes.** Changing criterion design used, stable responding achieved before increasing criterion.	**Yes.** Procedures and results are described using behavioral principles such as reinforcement, DRO, intervals, response effort etc.	**Yes.** Aim to increase face mask wearing in vulnerable populations during COVID-19 pandemic.	**Yes.** Clear behavioral definitions and detail on data recording procedures provided.
Halbur et al. (2021)	**Yes.** Generalization probes to face shield, generalization probes completed after treatment phase, multiple masks used/available throughout to promote generalization.	**Yes.** Face mask wearing/steps completed increased for all participants that completed the full study compared to baseline.	**Yes.** Procedures are described in enough detail for replication.	**Yes.** 5 dyads multiple probes baseline design, 1 dyad non- concurrent MBL design.	**Yes.** Procedures and results described in line with behavioral concepts such as reinforcement, discriminative stimuli, extinction, intervals etc.	**Yes.** Aim to increase face mask wearing during COVID-19 pandemic.	**Yes.** Behavioral definitions developed (though not included in paper), definitions provided for data collection and detail of data collection procedures available.
Hough (2022)	**Yes.** Generalization probes completed in the community.	**Yes.** Accuracy of mask wearing and duration increased above baseline levels for all participants.	**Yes.** Enough procedures are described in enough detail for replication.	**Yes.** Non-concurrent multiple baseline across participants	**Yes.** Procedures and interpretation of results in line with behavioural concepts such as behavioural skills training, positive reinforcement etc.	**Yes.** Aim to increase accurate face mask wearing and duration during COVID-19 pandemic.	**Yes.** Steps in hierarchy clearly defined along with adequate descriptions of data collect procedures.
Lillie et al. (2021)	**Yes.** Two generalization probes completed outside experimenter room, with two situations; walking and working.	**Yes.** Passive compliance for mask wearing increased for all participants.	**Yes.** Procedures are described in enough detail for replication.	**Yes.** Changing criterion design embedded in 4 tier non- concurrent MBL design.	**Yes.** Procedures and results are described using underpinning principles of behavior such as schedules of reinforcement, differential reinforcement etc.	**Yes.** Increasing passive mask wearing during COVID-19 pandemic.	**Yes.** Behavioral definitions developed (though not included in paper) along with clear descriptions/definitions of data collection procedures.
Sivaraman et al. (2021)	**Yes.** Two generalization probes completed with a novel mask and community setting.	**Yes.** Face mask wearing increased across all participants compared to baseline.	**Yes**. Procedures are described in enough detail for replication	**Yes.** Non-concurrent MBL design used with 3 tiers to allow functional relations to be identified.	**Yes.** Procedures and results described and hypothesized using behavioral principles such as reinforcement, graded exposure, prompt levels etc.	**Yes.** Aim to increase face mask wearing during global COVID-19 pandemic. Caregivers reported to have found the intervention useful and practical.	**Yes.** Behavioral definitions provided, clear descriptions of data collection procedures included.

### Search Strategy

**Fig. 1:**
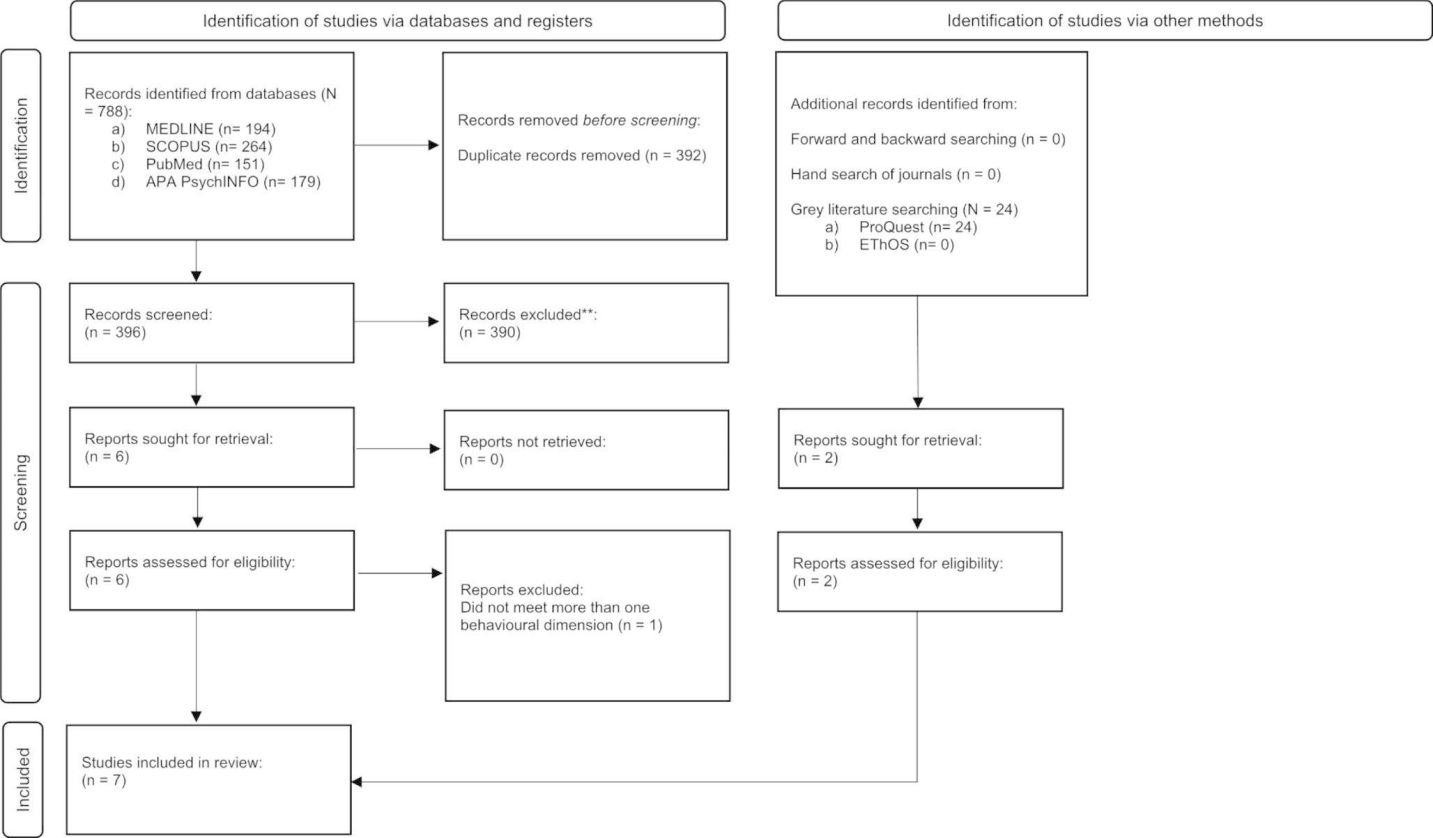
PRISMA Flowchart for Identifying Relevant Papers

Database searches of peer reviewed literature and grey literature were conducted in May 2023 (see Fig. 1). For peer reviewed literature we searched PubMed, MEDLINE, APA PsychINFO, and SCOPUS. For grey literature we searched ProQuest and EThOS. The search string used was autis* OR “autis* spectrum disorder” OR ASD OR ASC OR “autis* spectrum condition” OR PDD-NOS OR asperger* OR “development* disabilit*” AND “face mask*” OR “face cover*” OR mask. A total of 812 studies were identified through database searches, and following the screening of the titles and abstracts, eight studies were selected for a full-text review.

We hand-searched relevant journals to identify additional studies. The four journals were the (a) Journal of Applied Behavior Analysis, (b) Behavior Modification, (c) Behavioral Interventions, and (d) the International Journal of Positive Behavioural Support. No additional papers were identified. We reviewed the reference lists of studies selected for full-text screening (i.e., backward-searching) but did not identify additional studies. We also engaged in forward-searching through Google Scholar to identify articles that had cited the papers included in the full-text screening. Three papers were identified, but two were excluded due to not being empirical studies, and the other was excluded as it did not measure outcomes relevant to face mask-wearing.

This review was registered with PROSPERO, an international database of systematic reviews in health and social care (Registration Number: CRD42022296760).

### Screening

Eight papers were selected for full-text screening. Each of these articles was evaluated against the inclusion criteria and the seven dimensions of ABA to confirm they were behavior-analytic (see Table 1). We adapted this process from Lucock et al. (2019). Following full-text screening, one paper was excluded from the review (Aaronson et al., 2021) due to not meeting all seven dimensions of ABA. As a result, seven articles were included in the review.

### Risk of Bias Assessment

#### Evaluative Method

All included studies used single-case experimental designs (SCED). Therefore, we assessed their methodological quality using the evaluative method (Reichow et al., 2008; see Table 2). Studies were reviewed against primary (e.g., baseline, independent variable, visual analysis) and secondary (e.g., fidelity, IOA, Kappa) quality indicators and awarded an overall rating ranging from weak to strong. The Evaluative Method has been deemed the most robust in identifying studies’ weaknesses and distinguishing clearly between ‘weak’ and ‘adequate’ evidence (Wendt & Miller, 2012). It has also been shown to have the highest congruence to the quality indicators for SCED, as articulated by Horner et al. (2005).

**Table 2:** Data Extraction of Included Studies

Author (Year), Country, Quality Rating	Participant demographics (Number, sex, age, ethnicity, diagnosis, attrition)	Setting	Experimental design	Intervention; Implementer	Dependent variable (s)	Procedural fidelity	Generalization, Maintenance, Social Validity	Outcomes
Ertel (2020), USA, Borderline strong	**N =** 3 (All male) **Age range**: 4–8 **Ethnicity**: Not specified **Diagnosis**: All autistic **Attrition**: 0	Sessions took place across EIBI clinic, in participant’s homes, and in a mock physician’s office. Pre and post intervention probes were conducted in a hospital waiting room, and physician office	Concurrent multiple baseline across participants.	MSWO preference assessment, graduated exposure hierarchy, positive reinforcement, Experimenter for intervention phase, once mastered parents were then coached to implement procedures at home and in the community.	Compliance to steps in the exposure hierarchy. Steps completed reflected duration. Data was also collected on vocal protests, negative vocalisation, and mands for mask removal.	Treatment integrity data were collected on 65% of Patrick’s sessions, 76% of Chris’s sessions, and 76% of Cameron’s sessions. Mean treatment integrity was 100% for Patrick, 99.7% for Chris, and 99.3% for Cameron	**G**: Post-intervention probes were conducted in the waiting room and physician office settings. **M**: None **SV**: Caregivers answered two questions and collated open ended comments	All participants were able to tolerate the mask for at least one hour after treatment. Participants were able to tolerate the mask in the untrained settings.
Ertel et al. (2022), USA, Adequate	**N** = 3 (1 Female, 2 Male) **Age range**: 4–9 years old **Ethnicity**: Asian and Hispanic, Caucasian and Asian **Diagnosis**: All autistic **Attrition**: 0	Intervention sessions took place across an EIBI clinic, in participant’s homes, and in a mock physician’s office. Probes were conducted in another community setting.	Concurrent multiple baseline across participants.	MSWO preference assessment, graduated exposure hierarchy, positive reinforcement. Experimenter for intervention phase, once mastered parents were then coached to implement procedures at home and in the community.	Compliance to exposure hierarchy steps. Data was collected on compliance to the step and total duration of mask wearing during each trial. Data was also collected on negative vocalizations, and mands to remove the mask.	Treatment integrity data taken on a 60–85% of participant’s sessions. Mean treatment integrity for all participants was 100%. Treatment integrity data were collected across all settings.	**G**: Training conducted at home and mock physician’s office. Novel community setting probes completed. **M**: Probes conducted in the EIBI clinic 1-month post-treatment **SV**: Caregivers completed a nine question Likert scale ranging from 0 (not at all) to 5 (very much).	Following intervention all participants wore their mask for 1 h in each setting and for multiple hours during maintenance probes.
Frank-Crawford et al. (2021), USA, Weak	**N (total)** = 6 (1 Female, 5 Male), **N (included)** = 4 (1 Female, 3 Male), **Age range**: Overall 6–20, for included participants 6–14 **Ethnicity**: Not specified **Diagnosis**: All autistic & IDD **Attrition**: 0	All on inpatient unit. For three included participants sessions were conducted in a 3.2 m × 3.2 m activity room and for one included participants sessions took place across various locations of the inpatient unit.	Changing-criterion design.	Blocking, reinforcement, DRO, non-contingent access to preferred activities or competing stimuli. Inpatient behavioral team.	Duration (seconds) of mask compliance. Frequency of successful attempts to remove the mask, blocked attempts to remove the mask, and frequency of targeted problem behaviors.	Not collected	**G**: None **M**: Component analysis to assess which components were necessary to maintain the terminal mask compliance goal (only completed for two included participants) **SV**: None.	Increases in compliance with mask wearing were achieved with all participants; however, the terminal duration was not met for one participant.
Halbur et al. (2021), USA, Weak	**N** = 12 (All male). **Age range**: 4–10 years old **Ethnicity**: 10 Caucasian, 1 Hispanic, 1 South Asian **Diagnosis**: All autistic, 1 with ADHD and 1 with moderate ID. **Attrition**: 3	9 participants in their usual behavioral clinic only, 2 telehealth only, 1 split between telehealth and clinic. Telehealth sessions took place from an unspecified location.	Five dyads arranged according to multiple-probe design across participants, one dyad arranged as non-concurrent multiple baseline across participants.	Graduated exposure, prompts, differential reinforcement, and escape extinction; Therapist for 10, 1 parent, 1 partially parent and therapist, and one self-implemented through coaching.	Number of hierarchy steps tolerated, frequency of blocking and problem behavior. Duration of all sessions.	Taken on implementation of the procedures according to protocol. Taken on a minimum 33% of sessions for each participant. Average treatment integrity for each participant ranged from 95–99%.	**G**: Two to five face masks were included in treatment sessions, evaluated under different teaching conditions **M**: None **SV**: Survey completed with health providers prior to the study, asking about benefits and limitations to children wearing face coverings.	Results showed increases in compliance to wearing face masks for nine out of twelve participants. Improvements were also seen for two of three participants that were discontinued.
Hough (2022), USA, Weak	**N (total)** = 4 (3 Female, 1 Male) **N (included) =** 1 Female **Age range**: Overall 18–23, included was 19 **Ethnicity**: 2 Caucasian 2 African American **Diagnosis**: 1 autistic, 1 with Downs Syndrome, all with mild intellectual/developmental disability **Attrition**: 0	Baseline and training sessions took place online via Zoom and participants in private rooms in their house. Generalisation and maintenance online conducted via Zoom with the participant in the community.	Non-concurrent multiple baseline across participants	Behavioural skill training (included instructions, modelling, rehearsal, and delivery of corrective feedback), The primary investigator	Completion of steps to correct face mask wearing and the duration of face mask wearing	Taken on a minimum 33% of sessions for each participant. Average treatment integrity was 93% and ranged from 89–95%.	**G**: Probes completed in the community **M**: Probes completed twice a week for two weeks following generalisation probes **SV**: Likert scale survey given to participants	Mask wearing accuracy and duration improved for all participants above baseline levels, however this was inconsistent and did not meet CDC standards for mask wearing.
Lillie et al. (2021), USA, Borderline adequate	**N** = 6 (1 Female, 5 Male). **Age range**: 4–14 years old. **Ethnicity**: 5 White, 1 Hispanic. **Diagnosis**: All autistic **Attrition**: 0	In a private therapy 3 × 3 room within an ABA day-treatment centre.	Changing-criterion design embedded within a non- concurrent multiple baseline design across participants.	MSWO, free operant and concurrent chains preference assessments, DRO without escape extinction, positive and negative reinforcement, fading of DRO; Study experimenters.	Occurrences of problem behavior and removals of facemask. Percentage of trials with passive compliance and latency to errors were scored.	Collected for an average of 43.9% of session across participants and averaged 99.7% across all participants.	**G**: Generalization probes were completed in two other settings/situations (working/walking) simulating natural situations for face mask wearing **M**: Four- and eight-week probes completed for two participants that achieved mastery during baseline **SV**: None	Four participants met criteria within 40 sessions maximum. Compliance was generalized across novel setting. Two participants met criteria in baseline.
Sivaraman et al. (2021), Across multiple countries (telehealth), Strong	**N** = 6 (1 Female, 5 Male). **Age range**: 6–8 years old. **Ethnicity**: 1 Mixed, 2 Indian, 3 Hispanic. **Diagnosis**: All participants were autistic. **Attrition**: 0	Sessions were conducted via telehealth with the experimenter present in their home or office, and the participants present in their respective homes or therapy centers.	Non-concurrent multiple-baseline design across participants.	MSWO preference assessment, graded exposure hierarchy, in vivo modeling, prompting and positive reinforcement; Caregivers and/or therapists coached to deliver steps.	Number of hierarchy steps completed, duration of mask wearing, frequency of problem behavior (including attempts to block or remove masks) and percentage of oxygen-saturated hemoglobin in the blood.	Taken on caregivers’/therapists’ fidelity to coaching procedures. Averaged 100% for 5/6 caregivers, and 96.8% for 1/6 caregiver across at least 40% of sessions.	**G**: Probes completed for all participants using a different type of mask and in variety of indoor and outdoor settings **M**: None **SV**: Parent or therapist asked to complete an adapted form containing 6 items scored on a 5-point Likert scale based on training acceptability.	All participants wore a face mask for 10 min without exhibiting behaviors described as challenging. This generalized to a novel mask or community setting. Mask wearing did not affect the percentage of oxygen hemoglobin saturation.

*Note.* ADHD = attention deficit hyperactivity disorder, DRO = differential reinforcement of other behavior; EIBI = early intensive behavioral intervention; ID = intellectual disability, IDD = intellectual and developmental disability, MSWO = multiple stimulus preference assessment without replacement.

To further improve the sensitivity and contextual fit of the quality assessment tool, we adapted some of its indicators for this study. First, if a diagnosis of autism was stated in the study, we marked the relevant indicator as a yes without the need for stating the diagnostic assessment tool. Second, for interventionist characteristics, we expected information related to their training and years of experience. If this information was missing or was unclear, we marked the relevant indicator as a no. This decision was based on SCED standards highlighted by (Ganz & Ayres, 2018). Third, if a standardized score was not necessary, we treated the relevant indicator as not applicable instead of scoring it as a no. Therefore, the overall rating for that primary quality indicator was scored as acceptable rather than unacceptable if it met other required subdomains.

Many articles used multiple baseline design (MBL) variations, while some embedded a changing criterion design (CCD). We reviewed the latter as MBL designs when determining functional relations. We made this decision as the quality assessment tool was more suitable for analyzing MBL designs. In addition, we reviewed Frank-Crawford et al. (2021) as a Baseline-Intervention (A-B) design, as this was considered a more parsimonious approach. Quality assessments were only completed for participants meeting the inclusion criteria. As a result, two participants in Lillie et al. (2021) and three participants in Halbur et al. (2021) were excluded from the quality assessment due to unavailable intervention data.

Finally, we used the adapted overall quality ratings as outlined in Tomlinson et al. (2018), which allowed for more sensitive ratings of the papers. The tool was originally developed with three overall ratings, weak, adequate, and strong. The adapted version has five ratings, including borderline adequate and borderline strong.

#### Risk of Bias Tool

We also used the single-case design risk of bias tool (SCED RoB; Reichow et al., 2018). The SCED RoB tool reviews four types of bias: detection, performance, selection, and other sources of bias. Each type of bias is broken down into several domains, which are scored as either low, unclear, or high risk (see Table 3). The final domain (i.e., other sources of bias) is rated as either low or high risk. The SCED RoB has been used in a number of SCED systematic reviews to date (Beqiraj et al., 2022; Chawner et al., 2019; Germansky et al., 2020), providing additional insight into the validity of conclusions drawn from reviewed studies (see Tables 1 and 2).

**Table 3:** Summary Table for Risk of Bias Domains

	Selection bias		Performance bias		Detection bias				Other bias				
	Sequence generation	Participant selection	Blinding of personnel /participants	Procedural fidelity	Blinding of outcome assessment	Selective outcome reporting	DV variable reliability	Data sampling bias		Source of other bias			
Ertel (2020)	+	+	-	+	-	+	+	+	+				
Ertel et al. (2022)	+	+	-	+	-	+	+	+	+				
Frank-Crawford et al. (2021)	+	+	?	-	?	+	-	+	-	Data missing due to error and inconsistencies in the implementation of the independent variable.			
	**Selection bias**		**Performance bias**		**Detection bias**				**Other bias**				
	Sequence generation	Participant selection	Blinding of personnel /participants	Procedural fidelity	Blinding of outcome assessment	Selective outcome reporting	DV variable reliability	Data sampling bias		Source of other bias			
Halbur et al. (2021)	+	?	?	+	?	+	?	+	-	Inconsistencies in implementation of the independent variable.			
Hough (2022)	+	+	-	+	-	+	+	+	+				
Lillie et al. (2021)	+	+	?	+	?	+	+	+	+				
Sivaraman et al. (2021)	+	+	-	+	-	+	+	+	+				

*Note.* + indicates low risk of bias; - indicates high risk of bias; ? indicates unclear risk of bias, as included in Barton et al. (2015) and Reichow et al. (2018). The sequence generation domain was scored as low risk of bias for all included studies, as randomization would not be suitable for the experimental designs used in them.

### Data Extraction

We extracted information on participants’ characteristics (i.e., age, sex, ethnicity, diagnosis, and attrition), country and setting where each study took place, experimental design used, intervention components, intervention implementers, dependent variables, study outcomes, and measures of procedural fidelity, generalization, maintenance, and social validity (see Table 2).

### Meta-Analysis

Along with assessing the risk of bias and conducting the data extraction, we conducted a meta-analysis to evaluate further the magnitude of effects produced by the studies in this review. That way, it was possible to reach a more robust conclusion about the effectiveness of procedures in the current body of evidence. Raw data were extracted across all papers for each participant using the WebPlotDigitizer software, which has been found to have high levels of intercoder reliability and validity (Drevon et al., 2017).

#### Non-Overlap of all Pairs & Baseline Corrected Tau

Two effect sizes were calculated for each participant across all studies using online calculators (Tarlow, 2016; Vannest et al., 2016). The non-overlap of all pairs (NAP) was used to determine the effect the intervention had on face mask-wearing compared to baseline. It has been highly correlated with the R^2^ effect size index and has been found to produce effect sizes comparable to other overlap indices (Parker & Vannest, 2009). The NAP score, p-value, and 95% confidence intervals (CI) were recorded for each participant’s data (see Table 4). Baseline Corrected Tau (BCT) was calculated for participants (see Table 4) to compare the effect of the intervention compared to baseline while accounting for monotonic trend in baseline (Tarlow, 2017). BCT effect size, standard error, and whether the baseline was corrected were also recorded. For both effect sizes, small effects were between 0 and 0.65, medium effects were between 0.66 and 0.92, and large effects were between 0.93 and 1.

**Table 4:** Effect Sizes Calculated for all Participants

Author/Year	DV	Conditions compared	Participant	NAP	95% CI	BCT, *BSL Correction*	SE	R^2^, Slope
Ertel (2020)	Steps of hierarchy completed	Baseline to intervention and generalization probes	Patrick	0.93**	0.52–1	0.60, *No*	0.24	-
			Chris	0.85*	0.43–1	0.03, *No*	0.34	-
			Cameron	0.96**	0.56–1	0.56, *No*	0.23	-
Ertel et al. (2022)	Steps of hierarchy completed	Baseline to intervention and generalization/maintenance probes	Miles	0.94**	0.50–1	0.45, *No*	0.23	-
			Bennett	0.96**	0.50–1	0.54, *No*	0.25	-
			Vivian	0.96**	0.46–1	0.44, *No*	0.24	-
Frank-Crawford et al. (2021)	Duration of mask wearing	Baseline to intervention (TX only or TX combined with TX/DRO)	Garrett	0.70	0.36–1	0.24, *No*	0.24	0.63, 74.72
			Tobias	0.57	0.29–1	0.10, *No*	0.22	0.34, 41.91
			Wesley	0.80**	0.46–1	0.36, *Yes*	0.14	0.14, 1.47
			Eleanor	0.93**	0.54–1	0.39, *No*	0.20	0.70, 4.03
			Miles	0.95**	0.46–1	0.35, *No*	0.19	0.59, 31.50
Halbur et al. (2021)	Steps of hierarchy tolerated	Baseline to treatment and treatment extension	Carl	0.91*	0.42–1	0.37, *No*	0.24	-
			Elias	0.95***	0.67–1	0.56, *No*	0.17	-
			Harrison	1.00**	0.50–1	0.49, *No*	0.25	-
			Pete	1.00***	0.63–1	0.58, *No*	0.19	-
			Wendell	0.69	0.30–1	0.19, *No*	0.21	-
			Kevin	0.92***	0.59–1	0.51, *No*	0.19	-
			Allen	0.82*	0.39–1	0.53, *No*	0.30	-
			Nolan	0.95***	0.58–1	0.59, *No*	0.22	-
			Javier	0.85*	0.35–1	0.59, *No*	0.22	-
			Ryan	0.90***	0.58–1	0.38, *No*	0.16	-
			Malik	1.00*	0.35–1	0.84, *No*	0.29	-
Hough (2022)	Duration of mask wearing	Baseline to intervention and maintenance/generalization	Summer	0.97**	0.47–1	0.50, *No*	0.26	-
Lillie et al. (2021)	Percentage passive compliance	Baseline to intervention and generalization	Otis	1.00*	0.39–1	0.53, *No*	0.30	-
			Lucy	0.98**	0.49–1	0.47, *No*	0.23	-
			Roman	0.89**	0.53–1	0.59, *No*	0.22	-
			Rhett	0.92***	0.57–1	0.61, *No*	0.20	-
Sivaraman et al. (2021)	Percentage of hierarchy steps completed	Baseline to intervention and generalization	Thomas	0.98**	0.55–1	0.48, *No*	0.22	-
			Abhi	0.99***	0.65–1	0.56, *No*	0.91	-
			Jaun	0.99***	0.70–1	0.62, *Yes*	0.17	-
			Maria	0.97**	0.55–1	0.37, *No*	0.18	-
			Selva	1.00***	0.65–1	0.63, *No*	0.20	-
			Mateo	0.99***	0.69–1	0.61, *No*	0.17	-

*Note.* DV = Dependent variable; NAP = Non-Overlap of All Pairs; CI = Confidence Interval; BCT = Baseline Corrected Tau, BSL = Baseline; SE = Standard Error; R^2^ = R squared; CCD = Changing Criterion Design. R^2^ was only calculated for Frank-Crawford et al. (2021) as they used a CCD as their experimental design

p-values indicated by *, where * is < 0.05, ** is < 0.01 and *** is < 0.001

#### Least Squares Method Line of Best Fit

Moreover, we calculated the line of best fit using the least squares method to calculate the R^2^ using IBM SPSS Statistics software version 28, as Frank-Crawford et al. (2021) used a CCD in their study. Manolov et al. (2022) suggested that non-overlapping indexes are not recommended for CCDs, as due to the nature of the design, little overlap is to be expected. Therefore, it was considered prudent to add this calculation to our meta-analysis to account for the nature of this experimental design. However, NAP and BCT were still calculated for consistency.

Participants were not included in effect size calculations if they did not meet the inclusion criteria or if there were no intervention data. It is also important to note that for Halbur et al. (2021), only data points for face masks were included within the meta-analysis for consistency, as graphs included both masks and face shields. One participant from Halbur et al. (2021) was excluded from the meta-analysis due to only having face shield data points. Sivaraman et al. (2021) recorded multiple dependent variables. For consistency, we included the percentage of exposure hierarchy steps completed. Lillie et al. (2021) was treated as an MBL design, terminal probes were not included, and data points were added across each intervention criterion for effect size calculations consistent with other MBL and CCD papers included. Due to Lillie et al. (2021) using a changing criterion design embedded within a multiple baseline design it was not possible to calculate the line of best fit. For Frank-Crawford et al. (2021), two participants had an intervention phase, followed by an intervention plus DRO phase. We combined the data from both phases as an overall intervention phase for the analysis.

### Inter-rater Agreement

Each methodological step of full-text screening, quality assessments, and data extraction was double-coded by the first author, a postgraduate student in applied behavior analysis with six years of experience, and the second author, a doctoral-level board-certified behavior analyst with ten years of experience. There was only one disagreement between reviewers, which was resolved by checking and resolving the source of this.

## Results

### Participant Demographics, Setting, and Country

There were a total of 40 participants across all included papers, with only three who were excluded due to not being autistic and one excluded as they did not have any baseline or intervention data. Thirty-six participants were included in this review. All included participants were autistic, with additional diagnoses specified for 22% of participants. Specifically, one participant had a diagnosis of attention deficit hyperactivity disorder, and seven participants, a diagnosis of intellectual and developmental disability. Halbur et al. (2021) was the only study in which three participants did not complete the study. The most common ethnicity was Caucasian (37.5%), followed by Hispanic (12.5%). Ertel (2020) and Frank-Crawford et al. (2021) did not specify the ethnicity of their participants. All studies that specified ethnicity included participants from three different ethnic groups, except for Hough (2022) and Lillie et al. (2021), whose participants were from two ethnic groups. The mean age across participants was 8 years, with a range of 4–19 years. Only 5% of included participants were aged 18 or over.

The intervention was delivered for just over half of the participants in their usual ABA clinic setting (52.5%), followed by telehealth (22.5%) and an inpatient setting (15%). One participant from Halbur et al. (2021) received the intervention in their ABA clinic and via telehealth. All studies were completed in the USA, except for Sivaraman et al. (2021) who recruited participants from multiple countries via telehealth, including India, Mexico, Costa Rica, and Belgium.

### Experimental Design

All studies used SCED. Specifically, six studies used a variation of the MBL design across participants, including non-concurrent MBL (Hough, 2022; Sivaraman et al., 2021), multiple probe design and a non-concurrent MBL (Halbur et al., 2021), concurrent MBL (Ertel, 2020; Ertel et al., 2022), and a CCD embedded in a non-concurrent MBL (Lillie et al., 2021). Finally, one study used a CCD (Frank-Crawford et al., 2021).

### Intervention and Implementers

All studies used two or more intervention components, as reported in Table 2. The most common intervention components across studies were graded exposure hierarchy and positive reinforcement utilised in four studies, and differential reinforcement used in three. Only one study used escape extinction (Halbur et al., 2021). Additional supports, such as shaping (Sivaraman et al., 2021) and prompts (Halbur et al., 2021), were also incorporated in two studies. Hough (2022) was the only study to utilise behaviour skills training directly with the participants. All studies except Hough (2022) completed preference assessments to identify potential reinforcers for each participant.

Interventions were implemented by hospital staff in an inpatient unit (Frank-Crawford et al., 2021), study experimenters (Ertel, 2020; Ertel et al., 2022; Hough, 2022; Lillie et al., 2021), and caregivers or the individuals’ regular ABA therapist (Halbur et al., 2021; Sivaraman et al., 2021). In Ertel (2020) and Ertel et al. (2022), the intervention was completed by the study experimenters until mastery, and parents were then coached to implement procedures at home and in the community. In Halbur et al. (2021), one participant was coached to self-administer the intervention.

### Dependent Variables

The target behavior across all studies was an outcome relating to increasing tolerance to face mask-wearing. Primary measures reported across all studies were frequency of behavior described as challenging, frequency of mask removal or blocking, and duration of mask-wearing. Of all studies, five also recorded the number of exposure hierarchy steps completed (Ertel, 2020; Ertel et al., 2022; Halbur et al., 2021; Hough, 2022; Sivaraman et al., 2021). Two studies recorded compliance per session or trial (Ertel et al., 2022; Lillie et al., 2021), with Lillie et al. (2021) recording latency to error and Sivaraman et al. (2021) recording the percentage of oxygen-saturated haemoglobin in the blood using an oximeter.

### Procedural Fidelity

Six studies included procedural fidelity measures (Ertel, 2020; Ertel et al., 2022; Halbur et al., 2021; Hough, 2022; Lillie et al., 2021; Sivaraman et al., 2021). Each of these studies took fidelity data across a minimum of 33% of sessions and five of these found 99% or more fidelity when implementing procedures with only Hough (2022) scoring less than this with an average of 93% fidelity. In Sivaraman et al. (2021), fidelity data was only taken on caregiver implementation of coached steps and not on experimenters’ coaching of procedures.

### Maintenance, Generalization, and Social Validity

#### Maintenance

Four studies assessed the maintenance of intervention effects (Ertel et al., 2022; Frank-Crawford et al., 2021; Hough, 2022; Lillie et al., 2021). Ertel et al. (2022) completed maintenance probes one-month post-intervention for each participant and found results had maintained. Frank-Crawford et al. (2021) completed component analyses for 50% of included participants and demonstrated that duration of mask-wearing was maintained when removing intervention components. Lillie et al. (2021) completed four- and eight-week maintenance probes with the two participants that achieved mastery during baseline and found passive compliance remained at mastery level. Hough (2022) completed maintenance probes twice a week for two weekly post generalisation probes and found inconsistent results.

#### Generalization

Generalization was assessed in six studies (Ertel, 2020; Ertel et al., 2022; Halbur et al., 2021; Hough, 2022; Lillie et al., 2021; Sivaraman et al., 2021). Of these studies, four completed generalization probes across untrained settings (Ertel, 2020; Ertel et al., 2022; Hough, 2022; Lillie et al., 2021), one included multiple types of face masks and face shields during training sessions (Halbur et al., 2021), and one included both untrained settings and different mask types (Sivaraman et al., 2021). Four of these studies demonstrated that intervention effects had successfully generalized across untrained settings (Ertel, 2020; Ertel et al., 2022; Lillie et al., 2021; Sivaraman et al., 2021). In Sivaraman et al. (2021), four participants achieved tolerance of a novel face mask or setting for the entire 10-minute generalization probes, with only two participants tolerating just under (7 and 7.5 min) in their second generalization probe. Halbur et al. (2021) tested for generalization across mask types and face shields for 83% of participants and found varying levels of generalization.

#### Social Validity

Five studies assessed for social validity, three using a 5-point Likert scale with caregivers (Ertel, 2020; Ertel et al., 2022; Sivaraman et al., 2021), one completed a 5-point Likert survey with the participants (Hough, 2022), and one using a survey with healthcare professionals (Halbur et al., 2021). All results from social validity surveys reported acceptability for the procedures and satisfaction with the achieved outcomes.

#### Outcomes

For six studies, positive outcomes regarding increased mask-wearing were achieved across all participants (Ertel, 2020; Ertel et al., 2022; Frank-Crawford et al., 2021; Hough, 2022; Lillie et al., 2021; Sivaraman et al., 2021). Outcomes ranged from participants achieving target duration criteria of 5 min (Halbur et al., 2021), 10 min (Sivaraman et al., 2021), 10–60 min (Frank-Crawford et al., 2021), 30 min (Lillie et al., 2021) and 60 min (Ertel, 2020; Ertel et al., 2022). In Frank-Crawford et al. (2021), face mask tolerance increased for all participants throughout the intervention, with all but one participant achieving their terminal duration. In Halbur et al. (2021), nine participants achieved the target duration of face mask-wearing. For the remaining three participants that did not complete the intervention, two of these still showed some improvements in tolerating face masks following completing some steps of the exposure hierarchy. For Hough (2022) although improvements in duration and accuracy of mask wearing increased across participants compared to baseline levels, this did not maintain over time or generalise to a novel setting consistently, and overall performance decreased over time. Specifically, the one participant included in this review from this study found an increasing trend in correct trials and duration of masking wearing during intervention, reaching the maximum duration (10 min) and 100% accuracy by the end of the intervention phase, however performance reduced and became variable during generalisation and maintenance.

### Risk of Bias Assessment

#### Evaluative Method

The Reichow et al. (2008) tool was used to evaluate all studies. One paper was rated as strong (Sivaraman et al., 2021), one as borderline strong (Ertel, 2020), one as adequate (Ertel et al., 2022), one as borderline adequate(Lillie et al., 2021), and three were rated as weak (Frank-Crawford et al., 2021; Halbur et al., 2021; Hough, 2022).

#### Risk of Bias Assessment

The Reichow et al. (2018) SCED RoB tool was also used (see Table 3). The studies which had the highest number (seven out of nine) of domains scored as low risk were Ertel (2020), Ertel et al. (2022), Hough (2022), Lillie et al. (2021) and Sivaraman et al. (2021). Halbur et al. (2021) and Frank-Crawford et al. (2021) only had four out of nine domains scored as low risk. The most common domains rated as either high risk or unclear across all studies were blinding of personnel and participants and blinding of outcome assessment.

### Effect Sizes

A total of 33 effect sizes were calculated (see Table 4). Three participants were excluded, two participants due to having no intervention data (Lillie et al., 2021) and one due to not having intervention data on face mask-wearing (Halbur et al., 2021). The average NAP effect size across all studies was medium at 0.92 and for BCT small at 0.47. Of all studies, 57% had a large average NAP effect size, and 100% had an average small BCT effect. Sivaraman et al. (2021) had the highest overall average NAP effect size of 0.99 and BCT of 0.55 across participants, followed by Ertel et al. (2022) with 0.95 NAP and 0.48 BCT effects, and Lillie et al. (2021) with 0.95 NAP and 0.55 BCT. Halbur et al. (2021) and Ertel (2020) both had an overall medium NAP effect size of 0.91, with overall small BCT sizes of 0.51 and 0.40 respectively. Frank-Crawford et al. (2021) had the lowest effect sizes, with NAP showing a medium effect of 0.79 and BCT a small effect of 0.29. For Hough (2022) effect sizes were calculated for the one included participant with a large NAP effect size of 0.97 and small BCT effect of 0.50.

It is also important to note an outlier in Sivaraman et al. (2021) with a high standard error at 0.91 for one participant (i.e., Abhi), indicating results for this participant should be considered with caution. In addition, 95% CIs were wide, defined by a range of 0.5 to 1, for six studies. The average CI range was largest for (Frank-Crawford et al., 2021) at 0.58, followed by 0.53 for Hough (2022) and 0.51 for Ertel et al. (2022), and Halbur et al. (2021). This indicates the possibility of a wide margin of error for the effect sizes that should be considered when interpreting findings. However, it should be noted that no CIs crossed zero, which suggests that all studies had a positive effect.

### Line of Best Fit

R^2^ was calculated for the included participants in (Frank-Crawford et al., 2021) using the least squares line of best fit as a supplemental measure to assess the rate of change as the criterions progressed (Manolov et al., 2022). Three participants had high scores of 0.59, 0.63 and 0.70, indicating that the intervention had a more considerable effect. The remaining participants had low scores of 0.34 and 0.14, indicating that other variables may have influenced the duration of mask-wearing. As for the trend line slope, it was calculated as 1.47 and 4.03 for two participants indicating a low rate of change in mask-wearing across the intervention. For the other three participants, the slope was calculated at 31.94, 41.91 and 74.72, demonstrating a higher rate of change across sessions.

## Discussion

The current systematic review and meta-analysis reviewed behavior-analytic interventions for increasing face mask-wearing in autistic individuals. Overall, studies yielded positive outcomes with large or medium NAP effect sizes. Caution is needed, however, in interpreting findings as CI ranges were wide across six studies, and all studies had a small BCT effect size indicating baseline performance may have influenced the intervention effects. Exposure to face masks in baseline may have influenced performance and increased tolerance prior to the intervention phase. This was seen in Lillie et al. (2021), where two participants achieved mastery criteria in the baseline condition. For Frank-Crawford et al. (2021), the evaluation of the rate of change also showed variable findings across participants, which indicated findings may be related to issues with study design or confounding variables rather than being able to attribute these to the procedures themselves. Overall, increased tolerance of mask-wearing was achieved across all studies, indicating that behavioral analytic procedures are reasonably effective in building tolerance to face masks in autistic individuals. However, this is with consideration that the magnitude of effect sizes may not be certain and are likely influenced by issues with study quality or design.

We also examined the most common and effective intervention components, as all studies used multi-component intervention packages. The main intervention components were exposure hierarchies and differential and contingent reinforcement. These findings are similar to the ones by Allen and Kupzyk (2016), who reviewed procedures to increase compliance with medical and dental procedures in populations with IDD. Their findings demonstrated that most studies used multi-component interventions primarily based on graded exposure hierarchies and contingent reinforcement. In addition, a review by Jennett and Hagopian (2008) found that graded exposure and reinforcement were the most common components to treat phobias in individuals with IDD. They defined graded exposure as breaking down steps that progress chronologically (e.g., hierarchy) or increasing exposure to stimuli though changes in dimensions such as size, duration, or distance. By this definition, all included studies for the present review incorporated exposure, even if not all used a structured hierarchy by increasing the duration of mask tolerance over time.

No notable differences were found across studies with and without the use of an exposure hierarchy, though the majority of the studies with the largest effect sizes used exposure hierarchies and reinforcement as primary intervention components (Ertel et al., 2022; Halbur et al., 2021; Sivaraman et al., 2021). Moreover, in Lillie et al. (2021), the use of DRO without escape extinction showed comparably large effect sizes, although the study received a borderline adequate quality rating. It can also be noted that escape extinction was only used in Halbur et al. (2021) with little difference in outcomes compared to the other papers, meaning it is possible to increase face mask-wearing in autistic individuals without this intervention component. This is an important finding, as escape extinction can present several issues, such as impaired relationships, restricted autonomy, and increased risk of injury (Chazin et al., 2022). Regarding the generalization of mask-wearing, six studies provided generalization data (Ertel, 2020; Ertel et al., 2022; Halbur et al., 2021; Hough, 2022; Lillie et al., 2021; Sivaraman et al., 2021), and all but one (Hough, 2022) found that results were able to be transferred to novel settings and/or novel face coverings. This finding is key as face masks were required to be worn in a number of settings, such as supermarkets, libraries, schools, and public transport (Public Health England, 2020). More limited data were available to assess the maintenance of findings, as only two studies provided maintenance measures for all participants. One study reported positive results with performance maintained 1-month post-intervention (Ertel et al., 2022). For Hough (2022) they found their results did not maintain or generalise at a consistent level, which the author identifies to have been due to a lack of motivation in the absence of positive reinforcement rather than a skill deficit. Overall, more studies are needed to assess whether findings could be extended post-intervention to conclude the outcomes’ longevity from the present studies.

This review also examined what factors may influence the effectiveness of these procedures. Three studies (Halbur et al., 2021; Hough, 2022; Sivaraman et al., 2021) used telehealth during their intervention, while the remaining studies completed intervention sessions in person (Ertel, 2020; Ertel et al., 2022; Frank-Crawford et al., 2021; Lillie et al., 2021). There were no differences in outcomes between studies that used telehealth versus those completed in person. These findings are supported by other literature where telehealth has yielded comparable outcomes to in-person support (Wacker et al., 2013). In addition, telehealth holds numerous benefits, such as reaching families in different countries or hard-to-access areas, being more resource efficient, and considering the context of a global pandemic also has merit in reducing the spread of infection (Monaghesh & Hajizadeh, 2020; Tomlinson et al., 2018). Furthermore, there were slightly larger effect sizes, but no critical differences in study quality seen in studies where the experimenter implemented procedures compared to those implemented by a natural agent such as a parent or usual tutor. In addition, no differences were found in treatment fidelity based on who implemented procedures. Two studies recorded treatment fidelity data on the natural agent’s implementation of procedures (Halbur et al., 2021; Sivaraman et al., 2021), and four took data on experimenter implementation (Ertel, 2020; Ertel et al., 2022; Hough, 2022; Lillie et al., 2021). Overall, all studies with the exception of Hough (2022) achieved 100% fidelity. This finding holds important implications as interventions implemented by natural agents increases maintenance, generalization, and positive outcomes of procedures (Gerow et al., 2018).

The final research question concerned the social validity of procedures. For six studies, either a natural agent or a natural environment was used during the intervention and/or generalization phases, increasing the procedures’ social validity (Ganz & Ayres, 2018). Social validity findings from four of the studies suggest that caregivers and professionals found procedures valuable and acceptable. However, only two studies assessed caregivers’ views on procedures, and one solely gathered professionals’ views, providing limited data on procedures’ acceptability. Future research should use social validity measures more widely (Ganz & Ayres, 2018). In addition, only one of the included studies surveyed the participant’s perceptions of procedures, which future studies may consider expanding upon (Hough, 2022). Such an attempt could include communication aids, such as augmentative and alternative communication (AAC) or Talking Mats (Logan et al., 2017; Stewart et al., 2018). Finally, only one study coached one of the participants in accessing the intervention by themselves, which could be another means of increasing social validity that future research could expand upon (Halbur et al., 2021).

### Limitations

The first limitation of the current systematic review is that only seven studies were included in the final sample providing a limited range of findings. The lack of more studies on the topic could be attributed to the unexpected nature of the pandemic and the time it takes for studies to proceed to publication. At the time of writing this review, it has been over two years since COVID-19 began (WHO, 2022b). The small sample size also means we have not been able to run a moderator analysis, which should be considered in the future with a larger body of evidence. A second limitation is that only three included studies were rated as adequate or above, with four studies rated either borderline adequate or weak. Therefore, findings should be interpreted with caution. A third limitation is that only 5% of included participants were 18 or over, therefore studies and literature were primarily focused on autistic children. Future research should further consider how these procedures can support autistic adults building tolerance to medical equipment and procedures. This is especially important as autistic adults are more vulnerable to both medical and psychiatric conditions requiring intervention (Croen et al., 2015). A fourth limitation is that for one study (Hough, 2022) three participants were excluded as they were not autistic, however they did have other intellectual and developmental disabilities. Further research on this area should consider broadening the scope to include both autistic individuals and those with developmental and intellectual disabilities. A fifth limitation is that six of studies included in the present analysis were predominantly based in the USA, except for Sivaraman et al. (2021). It would be important to consider how this may limit how findings apply in other cultures, such as those where attitudes to face mask-wearing may differ. For example, in Asian cultures, face coverings were used to reduce the spread of illness prior to COVID-19 (Zhang et al., 2022). In addition, these findings may have a limited impact on countries such as the UK, where policies around face mask-wearing included a list of exemptions, which included autistic individuals (Public Health England, 2020). A sixth limitation is that findings to date only demonstrate outcomes of interventions targeting mask-wearing up to a target duration of one hour. This does not necessarily represent naturalistic durations of mask-wearing, which would be required to significantly reduce COVID-19 transmission (Ertel et al., 2022). A seventh limitation concerns the effect sizes used. Both NAP and BCT are non-parametric effect sizes which are less sensitive than parametric measures, however there is no agreement in the current literature regarding which effect sizes are best for SCED studies (Parker et al., 2011). BCT also has been found to have poor control for brief baseline phases (Tarlow, 2017). In addition, the line of best fit was used as recommended by Manolov et al. (2022) for CCDs, however this is susceptible to outliers which may skew the data (Tarlow, 2017).

### Considerations

Further consideration of these findings would be concerning the impact of contextual factors and additional stressors experienced by families and caregivers during the COVID-19 pandemic when mask mandates came into effect. Mutluer et al. (2020) describe how families of autistic children were under increased stress during this time, with many children being out of education. In addition, COVID-19 was found to have significantly impacted caregiver well-being and saw an increase in behaviors described as challenging, sleep issues, hypersensitivities, and appetite changes in autistic children (Mutluer et al., 2020). Surveys completed with individuals with IDD and their families about their experiences during the pandemic highlight other impacts such as social isolation, loss of usual activities, change and loss of routine, and reduced access to support services (Flynn et al., 2021; Peacock-Brennan et al., 2021). This highlights the importance of contextual fit when recommending or implementing behavioral interventions in high-stress situations alongside multiple competing variables. Furthermore, the benefits of wearing masks must be balanced with the potential negative impact. Mask-wearing in autistic individuals may lead to additional challenges related to the novelty of wearing a mask or the increase in face touching (Halbur et al., 2021, 2022). Face masks for children are also less likely to fit snugly to their faces which can reduce their effectiveness (Esposito & Principi, 2020). In addition, sensory needs may cause severe distress in some autistic individuals, and it may not be ethical or feasible to implement a mask-wearing procedure in such circumstances. In those cases, other alternative hygiene practices would need to be considered (Esposito & Principi, 2020). Face mask-wearing is generally recommended alongside other prevention strategies, such as washing hands and immunization, which should be taken into account with the present findings (Lio et al., 2021). These would be crucial factors for future policymakers to consider when developing mandates and guidance that may pose similar dilemmas for autistic individuals and their families.

### Implications

The findings from the current review go beyond face mask-wearing and extend current research on the use of behavior-analytic interventions to increase tolerance of medical equipment and hygiene practices in autistic individuals. Two years after the pandemic began, masks are no longer mandated in many countries such as the UK and USA, so it is essential to consider the broader implications of such findings to other tolerance-building programs for autistic individuals (Department of Health and Social Care, 2022; Tanne, 2022). This adds to a growing body of evidence for the use of desensitization programs with autistic and IDD populations to not only increase access to health care but also to reduce the use of physical and chemical restraints, which pose additional risks when used to access medical procedures (Babikian et al., 2020). These are crucial findings as the inability to access healthcare has a long-term impact on health and well-being, especially in autistic and IDD populations, where one in three deaths in adults in this population are due to preventable healthcare needs (Hosking et al., 2016).

Furthermore, to improve study quality in the future, it would be necessary for researchers to state interventionist characteristics in line with SCED standards (Ganz & Ayres, 2018). Six of the seven studies did not provide this information leading to a primary indicator being limited to an acceptable rating which impacts the overall study quality rating. Further clarity should be provided on the blinding of outcome assessors and study personnel to help reduce the risk of bias. However, readers should note that for many behavioral interventions, the experimenters and participants must be aware of the conditions for implementation (Germansky et al., 2020). It may also be beneficial to consider evaluating the effectiveness of these procedures in a larger study such as a randomized controlled trial to add to the existing body of evidence.

## Conclusion

In conclusion, the present findings show promising results for using behavior-analytic interventions to increase face mask-wearing primarily in autistic children, which adds to the current literature around increasing tolerance more broadly to medical devices, equipment, and procedures. However, these findings should be interpreted with caution and in consideration of the unique circumstance under which they were completed.

## Data Availability

Supplementary materials utilized during this review can be accessed at the FigShare data repository following this link: 10.6084/m9.figshare.22242775.v1.
